# Gene regulation by long purine tracks in brain related diseases

**DOI:** 10.1016/j.dib.2015.08.024

**Published:** 2015-09-04

**Authors:** Himanshu Narayan Singh, Moganty R. Rajeswari

**Affiliations:** a Department of Biochemistry, All India Institute of Medical Sciences, New Delhi 110029, India; b School of Sciences, Noida International University, Gautam Budh Nagar, 203201 Uttar Pradesh, India

**Keywords:** Purine repeat, Human genome, Gene regulation, Brain disease

## Abstract

Purine repeats are randomly distributed in the human genome, however, they show potential role in the transcriptional deregulation of genes. Presence of long tracks of purine repeats in the genome can disturb its integrity and interfere with the cellular behavior by introducing mutations and/or triple stranded structure formation in DNA. Our data revealed interesting finding that a majority of genes carrying purine repeats, of length *n*≥200, were down regulated and found to be linked with several brain related diseases [1]. The unique feature of the purine repeats found in the present study clearly manifests their significant application in developing therapeutics for neurological diseases.

Specifications table

**Table:** 

Subject area	Biology
More specific subject area	Genetics, Bioinformatics
Type of data	Table, Software generated files
How data was acquired	Software generated
Data format	Analyzed
Experimental factors	Purine repeats (*n*≥200) were searched in the human genome and also tried to explore their association with neurological disorders.
Experimental features	Purine repeat were searched by the help of home-made PERL script and further mapped them with neurological disorders
Data source location	New Delhi, India
Data accessibility	Data is supplied in this article

**Value of the data**

• Identified purine repeats (PR, *n*≥200) are unique in the human genome. Therefore, genes carrying purine repeats can be used as potential therapeutic tools in controlling gene expression and also in sequence-specific drug delivery.
• The data will be helpful to explore the risk associated with acquiring disease causing mutations related to diseases.
• The data will also be useful to study of evolutionary dynamics.

## 1 Data, experimental design, materials and methods

### 1.1 Data resources

In present study, four data resources were utilized viz. (i) Human Genome Sequence: NCBI/Genome database; (ii) gene annotation: Ensemble Genome Browser; (iii) gene-disease association: GenAtlas database; and (iv) expression datasets: NCBI/GEO database. Table 1

## 2 Algorithm developed for purine repeat search

An indigenous PERL script “PuRepeatFinder.pl” was developed to locate PRs, n≥200, in the human genome. The tool enlists the PRs in the chronological order of its genomic coordinates along with PR-length and sequence. The script implements the knowledge based window-shift algorithm, and identify only uninterrupted, non-overlapping purine repeats.

## 3 Web tools

We have utilized two web-tools: (i) non-B DNA Motif Search Tool (nBMST): to search for the mirror repeat motifs with the identified PRs. It searches for the perfect and imperfect mirror repeats within the provided sequences [2] and (ii) Idiographica: to show the distribution of PR-genes on the chromosomes [3].

## 4 Microarray data analysis

Two open source R-packages of Bioconductor project viz. limma: used for agilent based microarray data, and affy: for affymatirx based microarray data, were used to calculate gene expression levels. Expression computation involves three steps: (i) background correction, (ii) normalization and (iii) expression value computation [4]. Further, *t*-test was applied to screen statistically significant differential levels in mRNA expression of genes amongst patients and normal samples and *p*≤0.05 were considered as significant [1].

## Figures and Tables

**Table 1:** Description of PR-genes (polypurine nucleotides, *n*≥200) associated with neurological disorders, PR sequences and its coordinates in human genome. PR: Purine repeat.

**Gene symbol**	**Protein name**	**Contig**	**Chromosomal position**		**PR length**	**PR sequence**
**Start**	**End**
RABGAP1L	RAB GTPase activating protein 1-like	NT_004487.19	26199819	26200018	200	AAAAAAAAAAGAAGAAGAAGAAGAGGAAGAGGAGGGGGAGGGGGGAGGAGGAGGAAAGAAGAAGAAGAGGAGGAGGGGGAGGGGGAGGAGGAAGAAAGAAGAAGAGGAAGAGGAGAGGGAGGGGGAGGAGGAGGAAAGAAGAAGAAGAGGAGGAGGGGGAGGGGGAGGAGGAGGAAAGAAGAAGAAGAAAGAAAAGGGGG
ALK	anaplastic lymphoma receptor tyrosine kinase	NT_022184.15	8875666	8876076	411	GAAGAAGAAGAAAAGAAGAAGAAGAAGAAGAAAAGAAGAAGAAAAGAAGAAGAAGAAGAAGAAGAAGAAGAAGAAGAAGAAGAAGAAGAAGAAGAAGAAGGGGAAGAGGAAGGGGAAGGAGGAGGAGGAGGAGAAGGAGAAGAGGAAGGGGAGGAAGGGGAGGAGGAGGAGGAGGAGGAGGAGGAGGAGGAGGAGGAGAAGAAGAAGAAGAAGGGGAAGAAGGGGAAGAAGGGGAAGAAGGGGAAGAAGGAGAAGAGGAAGAGGAAGGGGAAGGGGAAGGGGAAGGGGAAGGGGAAGAGGAAGAGGAAGAGGAAGAAGAAGAGGAAGAAGAAGAGGAAGAGGAAGAGGAAGAAGAAGAAGAAGAAGAAGAAGAAGAAGAAGAAGAAGAAGAAGAAGAAGAAGAAGAAGAAA
GPR155	G protein-coupled receptor 155	NT_005403.17	25528765	25529048	284	GGGGAAAGAAGGAAGGAAGGAAGGAAGGAGGGAGGGAAGAAGGGAAGGAGGGAAGGAGGGAGGGAGGGAAGGAAGGGAAAGGAAGGAAAGGAAAGGAAGGGAAGAAAGGGAAGGGAAGGAAGGAAAAGGAAGGAAGGGAGAGGAAGGAAGGGAAAGGAAGGAAAGGGAAGGAAAGGGAAGGAAGGGAAGGGAAGGAAGGGAAAGGAAGGAAAAGAAAGGAAGGAAAAGGAAGGAAGGGAAGGAAGGGAAAGGAAGGAAGAGAAGGAAGGGAAAGGAAGGAAGGA
ROBO2	roundabout, axon guidance receptor, homolog 2 (Drosophila)	NT_022459.15	10576982	10577202	221	AAAAAAAAGAAAGAAGAGAAAGAAGAAAGAAAGAAGAAAGAGAAAGAAAGAAAGAAAGAAAGAAAGAAAGGGAGAGAAAGAAAGAAAGAAAAAGAAGAAAGAAAGAGAAAGAGAAAAAGAAAGAAAGAGAAAGAAAGAAAAAAAGAAGAAAGAAAGAAGGAAAGAAAGAAAGAAAGAAAGAAAGAAAGAAAGAAAGAAAGAAAGAAAGAAAGAAAAAAGAA
ARPP21	cAMP-regulated phosphoprotein, 21 kDa	NT_022517.18	35729714	35730024	311	AAAGGAAGGAAGGAAGGAAAAGAAAGAAAGAAAGAGAAAGGAGAGAGAGAAAGAAAGAAAAGGAGGAAGGAAGGAAGGAAGAAAGAAAGAAAGAAAGAAAAAGAAAGAAAGAAAGAAAAGAAAGGGAGAAAGGAGAGAGAGAAAGAAAAGGAAAGAAAGAAAGAAAAAGAAAAGAAAGGGAGAAAGGAGAGAGAAAGAAAAGAGAAAGAAAGAGAAAGAAAGAAAGAAAAGAAAGAAAAAGAAAGAGAGAGAGGGAGAGAGGGAGGGAAGGAAGGAAGAAAGGAAGGAAGGAAAGAAAGAGGAAAGAAAAG
35649333	35649547	215	AAAGAAGAAGAAGAAGAAGAAGAAGAAGAAGAAGAAGAAGAAGAAGAAGAGGAAGAGGAAGAGGAAGAGGAAGGAGGAGGAGGAGGAGGAGGAGAAGGAGAAGAAGAAAAGAAGAAGAAGAAGAAGAAGAAGAAGAAGAAGAAGAAGAAGAAGAAGAAGAAGAAGAAGAAGAAGAAGAAGAAGAAGAAGAAGGAGAAGAAGAAGAAAGAAGAAAG
APBB2	amyloid beta (A4) precursor protein-binding, family B, member 2	NT_006238.11	687107	687376	270	AAAAAAAAGGAAAGAAAGAAAAGAAAGAAAAGAAAGGAAAGAAAGAAAGAAAGAAAGAAAGAAAGAAAGAAAGAAAGAAAGAAAGAAAGAAAGAAAGAAGGAAGGAAGGAAGGAAGGAAGGAAAGAAAGAAAGAAAGAAAAGAAAGAAGGAAGGAAGGAAGGAAGGAAGGAAGGAAGGAAGGAAGGAAGGAAGGAAGGAAGGAAGGAAAGGAAAGGAAAGAAAGAAAGAAAGAAAGAAAGAAAGAAAGAAAGAAAGAAAGAAAGAAAGAA
JAKMIP1	janus kinase and microtubule interacting protein 1	NT_006051.18	4621548	4621869	322	AGGAGGGAAGGAAAGAAAGAAAAGAGAAAGAAAAGAAAGGAAAGGAAGGAAGGAAGAAAGAGAGAGAGAGAGAGAAAGAAAAAGAAAGAAAGAAAGAAAGAAAGAAAGAAAGAAAGAAGGAAAGGAAGAAAGAAAGGAAGAAAAGAAAGAAAAAGAAAAAGAAAGAAAGAAAAGAAAGAAAGGAAAGGAAAGGAAGAAAGAGAGAGAAAGAAAGAAAGAAAGAAAGAAAGAAAGAAAGAAAGAAAGAAAGAAAGAAAAAGAAAGAAAGAGAAGGAAGGAAGGAAGGAAAGAAAGAGAAAGAAAAGAAAGAAAGAAAGAAAGA
SEMA5A	sema domain, seven thrombospondin repeats (type 1 and type 1-like), transmembrane domain (TM) and short cytoplasmic domain, (semaphorin) 5 A	NT_006576.16	9343147	9343376	230	AAAAGAAGAAGGAAGGAAAGGAAAGGAAGGAAAGGAAAGGAAAGGAAAGGAAAGGAAAGGAAGGAAGGAAAGGAAAGGAAAGGAAAGGAAAGGAAAGGAAAGGAAAGGAAAGGAAAGGAAAGGAAAGGAGGGAAAGGAAGGGAAAGGAAGGGAAGGGAAAGGAAAGGAAAGGAAAGGAAAGGAAAGGAAAGGAAAGGAAAGGAAGGAAAGGGAAGGGAAGGGAAGGGAAG
OFCC1	orofacial cleft 1 candidate 1	NT_007592.15	9735376	9735631	256	GAAGAAGAAGAGGAGGAGAAGGAGGAAGAAGAAAAGAAGAAAAGGAAGAAGAAAGAAGAAGAAGAGGAGGAGGAGGAGGAGAAAGAGAAGAAGAAGAAGGAGGAGGAGGAGGAAGAGGAGGAGGAGGAGGAAGAGGAGGAGGAGGAGGAGGAGAAAAAGAAGAAGAAGAAGAAGAGAAGAAGAAGAAGAAGAGGAAGAAGAAGAGGAAGAGGAAGAAGAAGAAGAAGAAGAAGAAGAAGAAGAAGAAGAAGAAGAA
CLIP2	CAP-GLY domain containing linker protein 2	NT_007933.15	11798037	11798336	300	GAAAGAAAGAAAGAGAGAGAGAGAAAGAAGGAAGGAAGAAAGGAAGGAAAGGGAAGGGAAGGAAGGGAAGGAAAGGAAGGAAGGGAAAGAAAGGAAGGAAGGGAAGGAAAGGAAGGAAGGGAAGGAAGAGAAGAAGGAGAGAAAGAAAGAAGGAAGGAAAGGGAAGAGAAGGGAAGGAAGGGAAGGAAGAGAAGAAAGAAAGAGAAAGAAAGAAAGAAAGAAAAAGAAGGAAGGGAAGGGAAGGAAGGGAAGAAAGAGAGAGAGAAAGAAAGAGAGAGAGAGAGAGAGAAAGAAAGAGAA
CNTNAP2	contactin associated protein-like 2	NT_007914.15	7518448	7518720	273	AGGAAGGAAAGAAGGAAGGAAGGAAGGAAGGAAGGAAGGAAGGAAGGAAGGAAGGAAGGAAGGAAGGAAAGAAAGAAAGAAGGAAAGAAGGAAAGAAGGAAGGAAGGAGGGAAAGAAGGAAGGAGGGAAAGAAGGAAGGAGGGAAAGAAGGAAGGAAGGAAGGAAGGAAGGAAAGAAGGAAGGAAGGAAAGAAGGAAGGAAAGAAGGAAGGAAAGGAAGAAAGAGAGAAAGGAAGAAAGAGAGAAAGAAAGAGAGAAAGAAAGAAAGAAAGAA
CSMD3	CUB and Sushi multiple domains 3	NT_008046.16	27438784	27438993	210	AAAAGGAAAAGAAAAGAAAAGAGAAAAGAAAGAAAAAAGAAAAGAAGAGAGAAAAAAGAAAAAAGAAAGAAAAAGAAAGAAAGAAAGAAAGAAAGAAAAGAAAGAAAGAAAGAAAGAAAGAGAAAGAAGGAAGGAAGGAAGGAAGGAAGGAAGGAAGGAAGGAAGGAAAGAAGGAAGGGAGGGAAGGAGGAAGGGAGGGAAAGAGAGAGG
LINGO2	Leucine rich repeat and Ig domain containing 2	NT_008413.18	28179212	28179722	511	AGGAAGGAAAGAAGGAAGGAAAAAAGGAAGGGAGGAAGGGAGGAAGGAAGGGAGGGAGGAAGGGAGGGAGGGAGGGAGGAAGGAAGGAAGGGAGGAAGGAAGGAAGGAAGGGAGGGAGGAAGGAAGGAAGGGAGGAAGGAAGGGAGGGAGGGAGGAAGGGAGGGAGGAAGGAAGGGAGGGAGGAAGGAAGGGAGGGAGGAAGGAAGGAAGGGAGGGAGGAAGGAAGGGAGGGAGGAAGGAAGGAAGGGAGGGAGGAAGGAAGGGAGGGAGGAAGGAAGGAAGGGAGGAAGGAAGGAAGGGAGGGAGGAAGGAAGGGAGGGAGGGAGGGAGGGAGGAAGGAAGGAAGGGAGGAAGGAAGGAAGGAAGGGAGGGAGGAAGGAAGGAAGGGAGGAAGGAAGGGAGGGAGGGAGGAAGGGAGGGAGGAAGGAAGGGAGGGAGGAAGGAAGGAAGGAAGGGAGGAAGGAAGGAAGGGAGGAAGGAAGGGAGGGAGGAAGGAAGGAAGGAAGGAAGG
GRK5	G protein-coupled receptor kinase 5	NT_030059.13	71851179	71851395	217	AGGGAGAGAGGGAAGAAAGGAAGGGAGGGAGAGAGGGAGGGAGGAAGGGAGAGAGGAGGAAGGGAAGGAGGAAGGGAGGGAGAGAAGAAAGAGGGAAGAAAGGAAGGGAGGGAGAGAGGGAGGGAGGAAGGGAGAGAGGAGGAAGGGAAGGAGGAAGGGAGGGAGAGAGGGAGGGAGGAAGGAAGGGAAGAAGGAAGGGAGGAAAGAAGGGAGAAGG
SHANK2	SH3 and multiple ankyrin repeat domains 2	NT_167190.1	16166836	16167154	319	GGAGAGGAAGGGAGAGGAAGGGAGGGAGGGAGAGGAAAGGAGGGAGGGAGAGGAAAGGAGGGAGGGAGAGGAAAGGAGGGAGGGAGAGGAAGGAAGGGAGGGAGAGGAAGGAAGGGAGGGAGGGAGAAGGGAGGGAAGGGGAAGGAGGGAGAGGGAGGGAGGGAAGGAGAGGGAGGGAGGGAGAGGGAGGGAGGGAAGGAGAGGGAGGGAAGGAGAGGGAGGGAGGGAGAGGGAGGGAGGGAAGGAAGGAGGGAGGGAAGGAGGGAGGGAGAGGGAGGGAGAGGAAGGGAGGGAGGGAGGGAGAAGGAAGAGGGAGGGA
FEZ1	fasciculation and elongation protein zeta 1 (zygin I)	NT_033899.8	28925647	28925867	221	AAGAAAGAAAGAAAGAAAGAAAGAGAGAAAAGAAAGAAAGAAAGAAAGAAAGAAAGAAAGAAAGAAAGAAAGAAAGAAAGAAAGAAAGAAGGAAAGAAGGAAAGAAGGAAAGAAGGAAAGAAGGAAAGAAGGAAAGAAGGAAAGAAGGAAAGAAGGAAAGAAAGAAAGAAAGAGAGAAAGAAAGAAAGAAAGAAAGAAAGAAAGAAAGAAAGAAAGAAAGG
FLT1	fms-related tyrosine kinase 1	NT_024524.14	10027782	10028001	220	AAAAGAAAGAAAGAAAGAAAGAAAGAAAGAGAGAGAGAGAGAGAGAGAGAAAGAAAAGAAAGAAAGAAAGAAAGAAAGAAAGAAAGAAGGAAGGAAGGAAGGAAGGAAGGAAGGAAGGAAGGAAGGAAGGAAGGAAAGAAAGAAAGAAAGAAAGAAAGAAAGAAAGAAAGAAAGAAAGAAAGAAAGAAAGAAAGGAAGAAAGAAAGAAAGAAAGAAAGAA
FGF14	fibroblast growth factor 14	NT_009952.14	16068629	16068829	201	AAAAAGGAAGGAAGGAGGGAAGGAGGGAAAGGGAGGGAAAGGGAGGAGAAGGGAGGGGAAGGGAGGGGAAGGGAAGGGAAGGGAAGGGAAGGGAAGGAAGGAAGGAAGGAAGGAAGGAAGGAAGGAAGGAAGGAAGGAAGGAAGGAAGGAAAGAGGGAGGGAAGGAAGGAAGAAAAAAAAGGAAGGAAGGAAGGAAGGAAA
LRFN5	leucine rich repeat and fibronectin type III domain containing 5	NT_026437.12	23250692	23250941	250	AAGAAGGAAGGAAGGGAGGGAGGAAGGGAGGGAGGGAGGGAAGAAGGAAGGAAGGAAGGAGAAAAGAAAGAGAAAGAAAGAAAGAAAGAAAGAAAGAAAGAAAGAAAGAAAGAAAGAAAGAAAGAAAGAGAAAGAAAGAAAGAAAGGAAAGAAAGAAAGAGAAAGAAAGAAAGAAAGGAAAGAAAGAAAGAAAGAGAAAGAAAGAAAAAGAGAAAGAAAAAGAGAGAGAGGAAAGAAGGAAGGAAGGAAG
CACNG3	calcium channel, voltage-dependent, gamma subunit 3	NT_010393.16	24216588	24216849	262	GAGAGAAGGAAGGAAGGAAGGGAGGAAGGAAGGAAGGAAAAAGAGAAGGAAGGAAGGAAAAGAAAGAAGGAAAGAAAGAAAAAGGAAAGAAAGAAAGAAAGAAAAAAGAAAGAAAGAAAAGAAAGAAGAAAGAAAGAAAAAGAAAGAAGAAAGAAGGAAGGAAGGAGAGAGAGAGAAAGAAAAAGAAAGAAGAAGGAAGGAAGGAGAGAGAGAGAGAAAGAGAAAGAAGGAAAGAAAAGAAAGAAAGAAAGAAAGAAGAAAG
RBFOX1	RNA binding protein, fox-1 homolog (C. elegans) 1	NT_010393.16	7219727	7220117	391	AAGGGAGGGAGGGGGAAGAAGGAAGGGAGAGAGAGGGAGGAAGGGAGAGAGAGGAAGGAAGGGAGAGAGAGGGAGGAAGGGAGAGAGAGGAAGGAAGGGAGAGAGAGGGAGGAAGGGAGAGAGAGGGAGGAAGGGAGAGAGAGGAAGGAAGGGAGAGAGAGGGAGGAAGGGAGAGAGAGGGAGGAAGGGAGAGAGAGGGAGGAAGGGAGAGAGAGGGAGGAAGGGAGAGAGAGGGAGGAAGGGAGAGAGAGGGAGGAAGGGAGAGAGAGGGAGGAAGGGAGAGAGAGGGAGGAAGAGGGGAAGGAAGGAAGGGAGAGAGAGGAAGGAAAGGAGAGAGAGGAAGGAAGGGAGGGAGGGGAAGGAAAGGGGAGGGAAAGAAGGAAGGGAGAGA
CACNA1A	calcium channel, voltage-dependent, P/Q type, alpha 1 A subunit	NT_011295.11	4864225	4864512	288	AAAAGAAAAGAAAGGAAAAGAAAAGAAAAGAAGGAAGGAAGGAAGGAGAAAGAAGGAAAGAAAGAGAGAGAGAGAGAAAGAAAGGAAAGAAAGAAAGAAAGAAAGAAAGAAAGAAAGAAAGAAAGAGAAAAGAAAGAAAGAAAAGAAGGAAGAAGAGAGGAAGGAAGGAAAGGAAAGAAAGGAAAGAGAAAGGAAAAAGGAAGGAAGGAAAGAAAGGAAGGAAGAAGGGAGGGAGGGAAGGAGGGAAAGAGAAAGGAAAGGAAGGAAAGGAGGAAGGAAGGAAAGGAG
KLK6	kallikrein-related peptidase 6	NT_011109.16	23731139	23731457	319	GGAGAGAGAGGAGGAGGAAGAGGAGAAGGAGGAGGAAGAGGAGAAGGAGAGGAGGAAGAGGAGGAGGAAGAGGAGGAGGAGGAAGAGGAGGAGGAAGAGGAGAAGGAGGAGGAGGAGGAAGAGGAGGAGGAAGAGGAGAAGGAGGAGGAGGAAGAGGAGGAGGAAGAGGAGAAGAAGGAGGAAGAAGAGGAGGAGGAAGAGGAGGAGGAGGAAGAGGAGGAGGAGGAAGAGGAGGAGGAGAAGGAAGAGGAGGAGAAGAGGAGGAAAAGGAGGAGGAGGAAAAGGGGGAGGAGGAAGAGGAGGAGGAGGAAGAGGAGAA
CLDN14	claudin 14	NT_011512.11	23530957	23531530	574	AGGGAGGAAGGAAGGGAGGGAGGAAGGAAGGGAGGGAGGGAGGGAGGGAGGAAGGGAGGGAGGAAGGGAGGGAGGAAGGGAGGAAGGGAGGGAGGAAGGAAGGGAGGGAGGGAGGAAGGGAGGGAGGGAGGAAGGGAGGGAGGGAGGGAGGGAGGGAGGAAGGGAGGGAGGGAGGAAGGGAGGGAGGAAGGGAGGGAGGGAGGGAGGGAGGGAGGAAGGGAGGGAGGGAGGGAGGAAGGGAGGGAGGAAGGAAGGGAGGGAGGGAGGAAGGGAGGGAGGGAGGAAGGGAGGGAGGGAGGGAGGAAGGGAGGGAGGGAGGGAGGAAGGGAGGGAGGAAGGGAGGAAGGGAGGGAGGAAGGGAGGAAGGGAGGGAGGGAGGGAGGAAGGGAGGGAGGAAGGGAGGGAAGGAGGGAGGGAGGAAGGGAGGGAGGAAGGGAGGGAGGGAGGAAGGAAGGGAGGGAGGGAGGGAGGAAGGGAGGGAGGAAGGAAGGGAGGGAGGGAGGGAGGGAGGGAGGGAGGGAGGAAGGAAGGGAGGGAGGGAGGGAGGGAGGAAGGGAGGGAGGGAGGGAGGGAG
ATRX	alpha thalassemia/mental retardation syndrome X-linked	NT_011651.17	107971	108187	217	AGAAGGAGAGGGAGAGGGAGAGGGGGAAAGGGAGAGAGGGAGAGAGGGAGAGAGGGAGAGAGGGAGAGAGGGAGAGAGGGAGAGAGGGAGAGGGAGAGAGGGGAAGAGGGAGAGAGGGGAAGAGGGAGAGGGAGAGAGGGGAAGAGGGAGAGGGAGAGAGGGGAAGAGGGAGAGGGAGAGAGGGGAAGAGGGAGAGGGAGAGAGGGGAGGAGGGAGG
PCDH19	protocadherin 19	NT_011651.17	22884307	22884561	255	AAAAAAAGAAAAGAAAGAAAGAAAAAAGAAAAGAAAAGAAAGGAGGGAGGAGAAGGGAAGGGGAAAAGAGAGAGAGAAAGAGAAAAAAGGAAAGAAGGAAGGAAGGAGGGAGGGAAGGAAGGAAGGAAGGAAGGAAGGAAGGAAGGAAGGAAGGAAGGAAGGAAGGAAGGAAGGGAGAGAGAGAGAAAGAAAGAAAGAAAGAAAGAAAGAAAGAAAGAAAGAAAGAAAGAAAGAAAGAAAGAAAGAAAGGGAAAG
GRIA3	glutamate receptor, ionotropic, AMPA 3	NT_011786.16	6865112	6865411	300	GAAGGAAGGAAGGAAGGAAGGAAGGAAGGGAGGGAGGGAGGGAGGGAGGGAGGGAGGGAGGGAAAGAAAGAAAGAAAGAAAGAAAGAAAGAAAGAAAGAAAGAAAGAAAGAAAGAAAGAAAGAAAGAAAGAAAGAGAGAGAAAGAAAGAAAAAGAAAGAAAGAAAGAAAGAAAGAGAAAGAAGAGAGAGAAAGAAAGAAAAAGAGAGAAAGAAAGAAGAGAAAGAAAGAAAAAGAAAGAGAAAGAAAGAAGAAAGAGAGAGAAAGAAAAAGAAAGAGAAAGAAAGAAAGAAAGAGAGAAA
