# Supplementary material for: Gene regulation by long purine tracks in brain related diseases
Source: Data Brief. 2015 Sep 4;5:218–25. doi: 10.1016/j.dib.2015.08.024 (PMC4589756; doi:10.1016/j.dib.2015.08.024)

**Brief Description of steps with diagrams to run “PuRepeat.pl”**

To run the perl script “PuRepeat.pl”, please follow the following steps:

1. Place Perl Script (e.g. PuRepeat.pl) and Sequence File (e.g. NC_00021.fasta) in one folder (e.g. Homo Sapiens)
2. Open Command prompt and run the following commands
   1. perl PuRepeat.pl + press enter


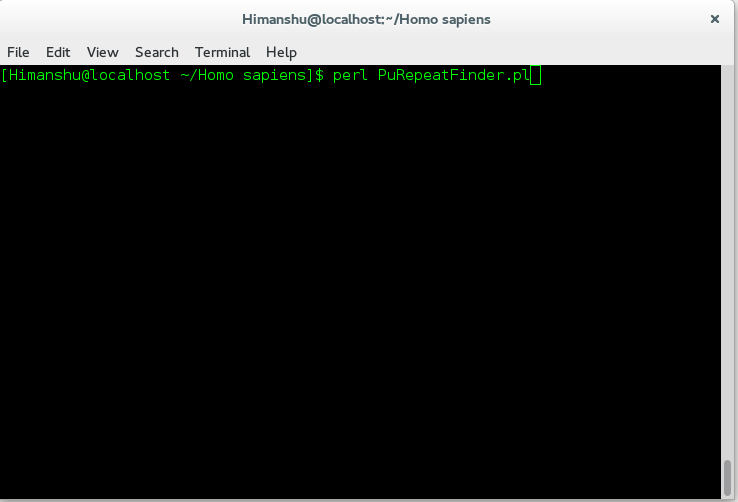


- 1. type the name of sequence file: NC_00021.fasta + press enter


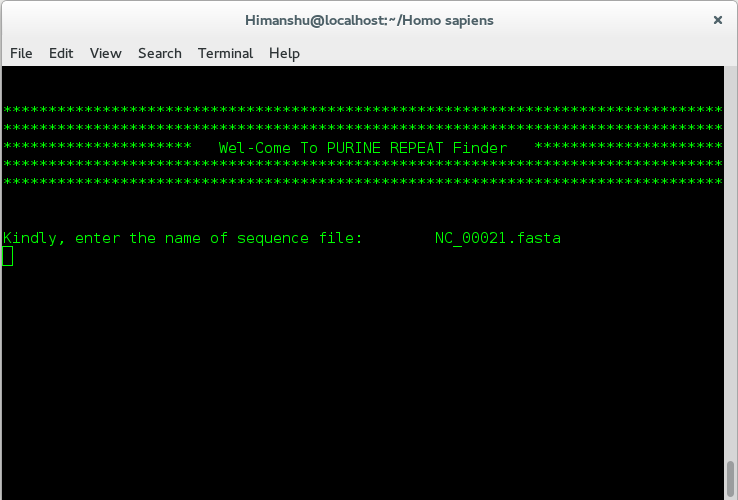


- 1. type minimum Purine Repeat Length: 200 + press enter


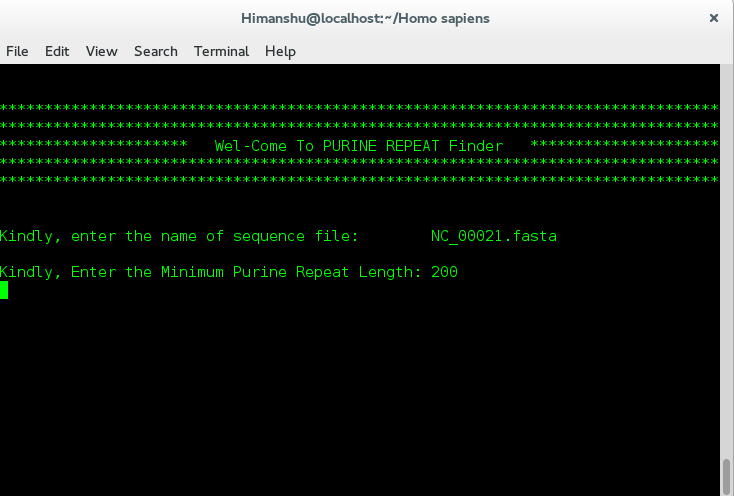


then process will be started with message “Please wait your file is being analyzed…”


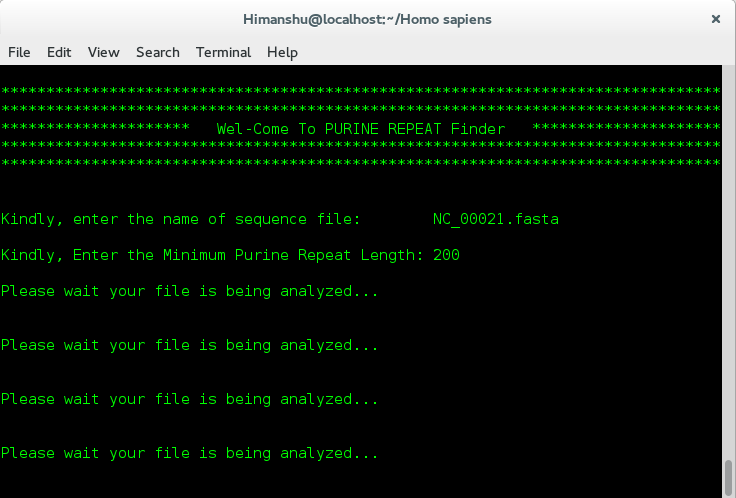


after completing of the job the PuRepeatRange.txt file will be generated which will be carrying

purine repeats with its coordinates and sequence.


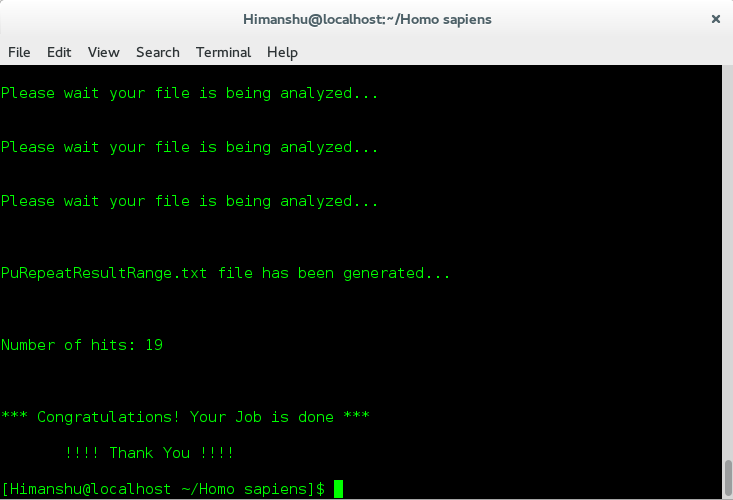

Supplement: Supplementary file 3 — Supplementary material [file mmc3.docx]
